# Ionically Crosslinked Complex Gels Loaded with Oleic Acid-Containing Vesicles for Transdermal Drug Delivery

**DOI:** 10.3390/pharmaceutics12080725

**Published:** 2020-08-02

**Authors:** Wing-Fu Lai, Ryan Tang, Wing-Tak Wong

**Affiliations:** 1 School of Pharmaceutical Sciences, Shenzhen University, Shenzhen 518060, China; 2016224040@email.szu.edu.cn; 2 School of Life and Health Sciences, The Chinese University of Hong Kong, Shenzhen 518172, China; 3 Department of Applied Biology and Chemical Technology, Hong Kong Polytechnic University, Hong Kong, China; w.t.wong@polyu.edu.hk

**Keywords:** transdermal delivery, vesicles, gel, transdermal flux, skin permeation

## Abstract

Skin is an attractive site for drug administration partly because of its easy accessibility and favorable properties (e.g., less invasiveness and high patient compliance) over some other common routes of administration. Despite this, the efficiency in transdermal drug delivery has been largely limited by poor skin permeation. To address this problem, this study reports the generation of oleic acid-containing vesicles, which can enhance the drug delivery efficiency while showing good stability and limited skin disruption. Upon being loaded into a complex gel, along with the incorporation of the polymer blending technique, a delivery system exhibiting tunable transdermal flux of 2,3,5,4′-tetrahydroxystilbene 2-O-β-D-glucoside is reported. Taking the good biocompatibility and tunable delivery performance into account, our system warrants further development and optimization for future applications in the treatment of skin diseases.

## 1. Introduction

Skin makes an attractive site for drug delivery partly because of its easy accessibility and favorable properties over other common routes of administration. For instance, compared to intravenous administration, transdermal drug delivery is less invasive and hence, results in higher patient compliance. Compared to using the oral route, delivering drugs transdermally can also avoid drug inactivation or degradation which may occur in the gastrointestinal tract. Transdermal drug delivery, however, has yet to fully achieve its potential as a drug administration method alternative to hypodermic injection at the moment. This is particularly true for hydrophilic drugs, in which the efficiency in transdermal drug delivery is largely affected by their inability to enter the skin at a therapeutically useful rate [1].

To enhance the efficiency in transdermal drug delivery, over the years, various strategies have been developed, ranging from electroporation [2,3] to the use of microneedles [4,5,6]. Compared to physical means which necessitate the availability of relevant devices for transdermal drug administration, chemical strategies simplify the execution process and make the process more easily applicable in practice. In this study, we report oleic acid-containing vesicles as an enhancing system for transdermal drug delivery. Our results demonstrate that upon the use of the vesicles as carriers, the transdermal flux of a drug can be substantially increased. Apart from high efficiency in skin permeation, high flexibility of the delivery system is desired as it can enable the system to be tuned to meet the practical needs of different treatment options. Previously, we have adopted a polymer blending technique to generate a gel system for controlled co-delivery of multiple drugs, with the release rate of each of the co-delivered drugs being able to be fine-tuned to meet the practical needs of a therapeutic regimen [7,8]. Here we take advantage of the close relationship between the composition and swelling/erosion behavior of our gel and integrate the gel with the vesicles to generate a biocompatible system which enables the transdermal flux of 2,3,5,4′-tetrahydroxystilbene 2-O-β-D-glucoside (THSG) to be manipulated. THSG is a water-soluble active component extracted from the dried tuber root of *Polygonum multiflorum* [9], which is a herb widely used in traditional Chinese medicine to treat skin depigmentation diseases [10]. The mechanism adopted by THSG to tackle the diseases has recently been found to link to the effect of THSG on upregulating the expression of microphthalmia-associated transcription factor (MITF) (which is needed for tyrosinase expression) and on activating cyclic adenosine monophosphate (cAMP) response element binding protein (CREB) [9], resulting in an increase in the melanin content and tyrosinase activity in a concentration-dependent manner in skin cells [9]. THSG is, therefore, a drug candidate that shows potential to be further developed into a transdermal formulation for real applications.

## 2. Materials and Methods

### 2.1. Materials

Calcium chloride (CaCl_2_) and sodium alginate (Na-Alg, Mw ≈ 20–50 kDa) were purchased from Macklin (Shanghai, China). THSG and carboxymethylcellulose sodium (CMC-Na, average Mw ≈ 10 kDa, degree of substitution = 0.7) were obtained from Sigma-Aldrich (St. Louis, MS, USA). Dulbecco’s modified Eagle’s medium (DMEM; Gibco, Grand Island, USA), fetal bovine serum (FBS; Hangzhou Sijiqing Biological Engineering Materials Co., Ltd., Hangzhou, China) and penicillin G-streptomycin sulfate (Life Technologies Corporation, USA) were used as the cell culture medium. Trypsin-ethylenediaminetetraacetic acid (EDTA) (0.25% trypsin-EDTA) was purchased from Invitrogen (Grand Island, NY, USA).

### 2.2. Synthesis of Oleic Acid-Containing Vesicles

32 µL of oleic acid and 4.2 µL of glycerol monooleate were mixed with 3 mL of chloroform. The solvent of the mixture was removed under reduced pressure using a rotary evaporator (Shanghai Qingpu Huxi Instrument Factory, Shanghai, China) at 55 °C to obtain a dry lipid film on the wall of a flask. Evaporation was carried out for additional 3 h after the appearance of the dry residue in order to completely remove the organic solvent. 1 mL of 1M Tris-HCl buffer (pH 8.0) containing a known amount of THSG (16 mg mL^−1^) was added to the film for hydration under vigorous stirring for 30 min. The vesicles produced were designated as V-THSG). They were stored at 4 °C for subsequent use. The same method was applied to produce blank vesicles but THSG was not added during the preparation process.

### 2.3. Determination of Size Distribution

The mean size and the size distribution of the vesicles were determined by using an optical microscope equipped with a digital camera (CCD-1, JVC, Tokyo, Japan). 500 vesicles in randomly selected views with a magnification of 40× were analyzed using Image J (National Institute of Mental Health, Bethesda, MD, USA).

### 2.4. Generation of a Vesicle-Loaded Complex Gel

An aqueous solution containing an appropriate mass-to-mass ratio of Na-Alg and CMC-Na was added into a collection bath containing a 10% (w/v) solution of CaCl_2_. The gel was formed and collected after 1 h of gelation at ambient conditions. The gel was designated as AC100, AC75, AC50 or AC25, with the number designating the mass percentage of Na-Alg in the Na-Alg/CMC-Na blend. After gel formation, the gel was lyophilized. 30 mL of a vesicle-containing solution was added to 1 g of a lyophilized gel. After incubation at 37 °C for 24 h, the vesicle-loaded gel was retrieved. The AC100, AC75, AC50 and AC25 gels were designated as AC100/V, AC75/V, AC50/V and AC25/V respectively, after being loaded with blank vesicles.

### 2.5. Mechanical Strength and Rheological Measurement

The viscosity of a complex gel, before and after the vesicle loading process, was examined using the Brookfield DV-III Ultra programmable rheometer (Brookfield Engineering Laboratories Inc., Middleboro, MA, USA) with spindles (CP-40). Viscosity parameters were collected at different shear rates under ambient conditions. The equilibration time at every shear rate was set to be 15 s. Viscoelastic properties of the sample were studied in the stress range of 0–100 Pa. The storage modulus (G′) and loss modulus (G″) of the sample were determined.

### 2.6. Determination of the Swelling and Erosion Behavior

A lyophilized and pre-weighed complex gel (0.05 g) was immersed in 100 mL of simulated body fluid. The gel was retrieved by centrifugation at a relative centrifugal force of 4000× *g* for 5 min, followed by the removal of the supernatant, at a predetermined time interval. The water absorption ratio (*WAR*) of the gel was calculated using the following formula:

(1) $$WAR=\frac{m_{s}-m_{d}}{m_{d}}$$

where m_s_ and m_d_ represent the mass of the swollen gel and the mass of the dried gel, respectively. To evaluate the erosion behavior, a known initial dry mass of a lyophilized gel was immersed into simulated body fluid, and was incubated at 37 °C. The sample was retrieved at a predetermined time interval, and was dried in an oven at 50 °C. The ratio between the final dry mass (m) and the initial dry mass (m_0_) was determined.

### 2.7. Cytotoxicity Assay

3T3 mouse fibroblasts, HDF cells and HaCaT cells were purchased from American Type Culture Collection (Rockville, MD, USA), and were cultured in DMEM supplemented with 100 UI mL^−1^ penicillin, 100 μg mL^−1^ streptomycin, 2 mM L-glutamine and 10% FBS. 24 h before the assay, cells were plated in flat-bottomed 96-well plates at a density of 5000 cells per well. The plates were incubated at 37 °C under a humidified atmosphere of 5% CO_2_. During the experiment, the cells were incubated with an appropriate amount of either the vesicles or the complex gel for 5 h at 37 °C. After that, the medium in the plates was replaced with the fresh cell culture medium. The CellTiter 96 AQueous non-radioactive cell proliferation assay (MTS assay; Promega Corp., Madison, WI, USA) was performed, according to the manufacturer’s instructions, either immediately or after incubation of the cells for additional 24 h to determine the cell viability (%) in each well.

### 2.8. Determination of the Encapsulation Efficiency (EE) and Loading Efficiency (LE)

V-THSG was prepared as mentioned above. The solution containing the vesicles was subjected to centrifugation at a relative centrifugal force of 10,000 × *g* for 1 min. The concentration of THSG in the supernatant was determined at 320 nm using a UV/Vis spectrophotometer (Varian, Inc., Palo Alto, CA, USA). The EE and LE were calculated using the following formulae:

(2) $$\mathrm{EE}\;\left(\%\right)=\frac{m_{T}-m_{F}}{m_{T}}\times 100\%$$

(3) $$\mathrm{LE}\;\left(\%\right)=\frac{m_{T}-m_{F}}{m_{G}}\times 100\%$$

where $m_{T}$ is the total mass of THSG added during the drug loading process, $m_{F}$ is the mass of THSG in the supernatant and $m_{G}$ is the dry mass of V-THSG. A similar method was also adopted to evaluate the EE and LE of the complex gel. In brief, 30 mL of a solution containing V-THSG was added to 1 g of a lyophilized gel. After incubation at 37 °C for 24 h, a vesicle-loaded gel was obtained. The concentration of THSG in the remaining vesicle-containing solution was determined at 320 nm using a UV/Vis spectrophotometer. The EE and LE were calculated by using formulae (2) and (3), where $m_{F}$ is the mass of THSG in the remaining solution and $m_{G}$ is the dry mass of the vesicle-loaded gel.

### 2.9. Skin Preparation

The skin was prepared according to a method previously described [11]. In brief, porcine ears were purchased from a local slaughterhouse. A clipper was used to remove hairs from the skin. Blunt dissection was performed to excise the full-thickness dorsal skin. The skin was immersed in a 0.05% (*w*/v) aqueous solution of sodium azide for 5 min and was stored at −20 °C for subsequent use.

### 2.10. Skin Permeation Analysis

The skin permeability of THSG was evaluated using a TP-6 Franz diffusion cell (Tianjin Jingtuo Instrument Technology Co., Ltd., Tianjin, China). During the experiment, the diffusion cell was first set at 37 °C. The receiver chamber was filled with 15 mL of a phosphate-buffered saline (PBS) solution (pH 7.4). The skin was slowly thawed, cut into the dimension of 2 × 2 cm, and fixed in the diffusion cell by enabling the dermal side to be in contact with the receiver medium and the epidermis side to be in contact with the donor chamber (with the contact area being 1.77 cm^2^). After the diffusion cell was clamped, the receiver medium was set to be under constant magnetic stirring at 600 rpm. 1 mL of a solution containing 40 mM of THSG was added to the donor chamber. At appropriate intervals, 300 μL of the receptor medium was collected and replaced with an equal volume of fresh PBS. The amount of THSG passing through the skin was determined using a UV/Vis spectrophotometer. The flux of THSG was calculated at the end of the experiment (24 h). The same method was also used to determine the skin permeability of V-THSG, but the THSG solution in the donor chamber was replaced with a solution containing V-THSG or a complex gel loaded with V-THSG. The total concentration of THSG in the vesicle-containing solution or vesicle-loaded gel was 40 mM.

### 2.11. Transepidermal Water Loss (TEWL) Examination

A TP-6 Franz diffusion cell was adopted to study the TEWL of the skin after treatment with the oleic acid-containing vesicles. Upon administration of a solution containing blank vesicles (or 1M Tris-HCl buffer (pH 8.0) as the control) for 5 h, the donor compartment was removed. A Tewameter^®^ 300 evaporimeter probe (Courage and Khazaka, Germany) was put on the skin surface to calculate the TEWL values in the unit of g m^−2^ h^−1^.

## 3. Results and Discussion

### 3.1. Characterization of Oleic Acid-Containing Vesicles as Transdermal Carriers

**Figure 1:**
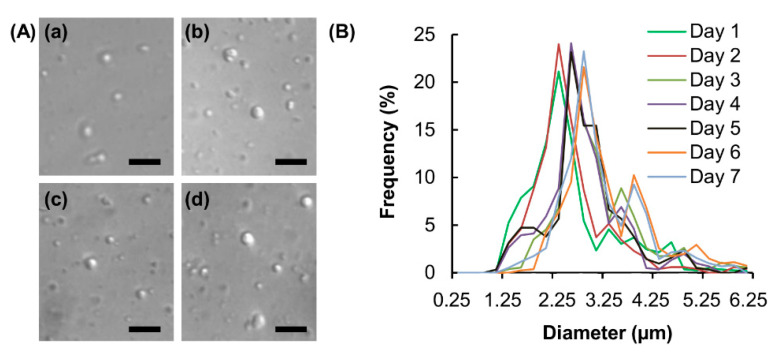
(**A**) Microscopic images of blank vesicles taken (**a**) 1, (**b**) 3, (**c**) 5 and (**d**) 7 days after the preparation process. Scale bar = 10 μm. (**B**) The size distribution profiles of blank vesicles sampled at different time intervals after vesicle fabrication.

**Figure 2:**
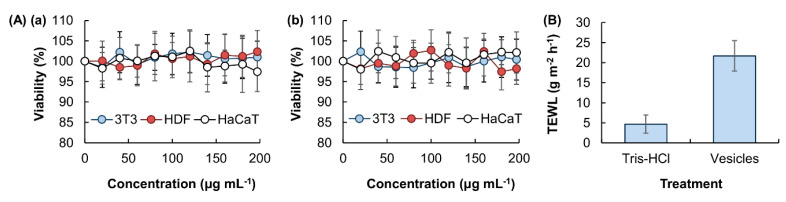
(**A**) The viability of 3T3, HDF and HaCaT cells after 5 h treatment with blank vesicles, (**a**) without or (**b**) with 24 h post-treatment incubation prior to the MTS assay. Data are presented as the means ± standard deviation (SD) of triplicate experiments. (**B**) TEWL values of a vesicle-containing solution and 1M Tris-HCl buffer. Data are presented as the means ± SD of triplicate experiments.

Oleic acid-containing vesicles are generated via the method of thin-film hydration. The average diameter of the generated vesicles is approximated to be 2.35 ± 0.9 μm (Figure 1), which enables the vesicles to be used subsequently as a skin permeation enhancer. The high safety profile of the vesicles in transdermal drug administration is evidenced in the MTS assay performed in different skin cell lines (Figure 2A). No apparent loss of cell viability, and hence no acute cytotoxicity, is observed after 5 h treatment with the vesicles. To evaluate possible chronic cytotoxicity, the viability of the treated cells is also examined after 24 h post-treatment incubation. No detectable loss of cell viability is noted in all vesicle concentrations tested. This reveals the high safety profile of the vesicles for subsequent use in transdermal drug administration.

**Figure 3:**
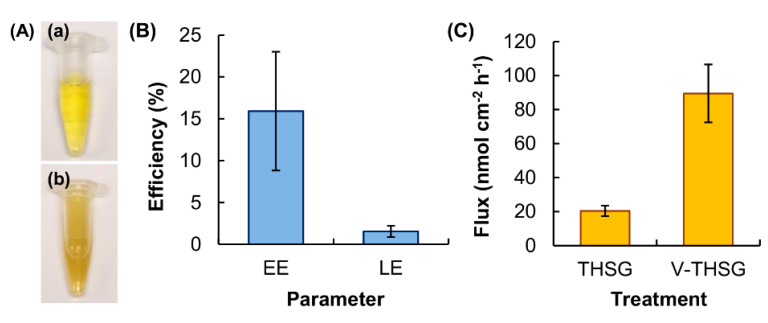
(**A**) Images of solutions of (**a**) THSG and (**b**) V-THSG. (**B**) The EE and LE of the vesicles. Data are presented as the means ± SD of triplicate experiments. (**C**) The transdermal flux of THSG from the plain THSG solution and the V-THSG solution. Data are presented as the means ± SD of triplicate experiments.

The TEWL value of the skin treated with the vesicles is five times higher than that of the non-treated skin (Figure 2B). This suggests that the vesicles may cause disruption of the skin barrier. Such an observation is consistent with the findings of earlier studies [12,13,14], which have reported that oleic acid can disrupt the skin barrier by dissolving the lipid chain of the stratum corneum. By using a model stratum corneum membrane containing bovine brain ceramide, cholesterol and palmitic acid, Rowat and co-workers have also found that oleic acid can promote phase separation in the membrane, leading to changes in the structure and permeability of the stratum corneum [15]. Along with the fact that oleic acid can enhance skin permeation by stimulating epidermal lipid bilayer fluidization and corneocyte shrinkage via keratin condensation [16], leading to the enlargement of aqueous pores for transdermal drug delivery [16], it is expected that the oleic acid-containing vesicles can facilitate the transport of hydrophilic drugs across the skin. To examine the efficiency of the vesicles as skin permeation enhancers, THSG is adopted as a hydrophilic drug model (Figure 3A). The EE and LE of the vesicles are approximated to be 15.9% and 1.6%, respectively (Figure 3B). Compared to plain drug, the use of the vesicles as carriers enhances the transdermal flux of THSG by around 4-folds (Figure 3C).

### 3.2. Properties of the Complex Gels for Sustained Vesicle Release

**Figure 4:**
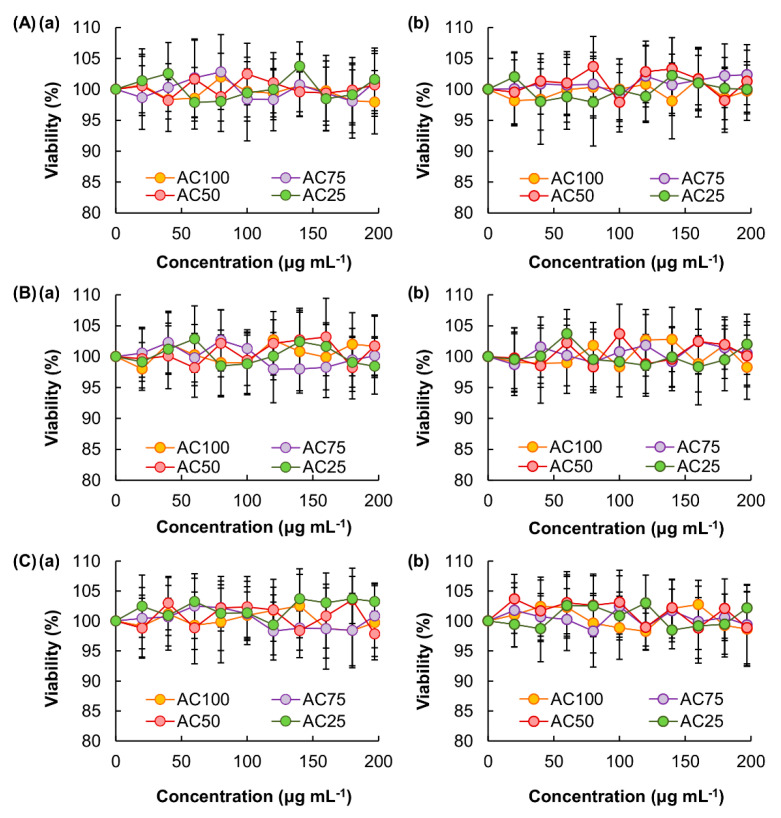
The viability of (**A**) 3T3, (**B**) HDF and (**C**) HaCaT cells after 5 h treatment with different complex gels, (**a**) without or (**b**) with 24 h post-treatment incubation prior to the MTS assay. Data are presented as the means ± SD of triplicate experiments.

To manipulate the transdermal flux of V-THSG, a complex gel formed between Na-Alg and CMC-Na is applied. Na-Alg is a naturally occurring polysaccharide comprising mannuronic (M) and guluronic (G) acid residues [17], with the M-blocks and G-blocks interspersed within regions of alternating structures [17]. Owing to its high biocompatibility and high abundance in nature, Alg-based materials have been generated in multiple forms, ranging from fibers to nanoparticles [18,19], for drug delivery over decades [20,21,22,23]. On the other hand, CMC-Na is a cellulose derivative commonly used as a food additive and has a track record of biomedical use in the literature [24,25,26,27,28]. Because both Na-Alg and CMC-Na are non-toxic and biodegradable polymers that have been widely used in food applications [29,30,31], the complex gels generated in this study are safe for clinical use. The lack of cytotoxicity of the polymers is confirmed by using the MTS assay in 3T3, HDF and HaCaT cells. Both the acute and chronic cytotoxicity of complex gels with different mass percentages of CMC-Na are found to be negligible (Figure 4).

**Figure 5:**
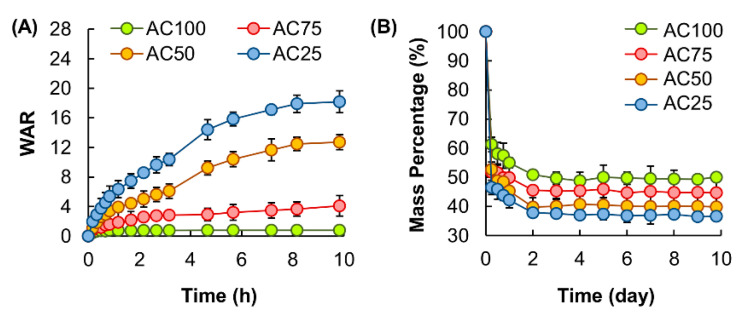
(**A**) The swelling capacity of complex gels with different mass percentages of CMC-Na in simulated body fluid. Data are presented as the means ± SD of triplicate experiments. (**B**) Susceptibility of complex gels with different mass percentages of CMC-Na to erosion in simulated body fluid. Data are presented as the means ± SD of triplicate experiments.

As far as vesicle release from the complex gel is concerned, water in the gel matrix is the medium through which vesicles diffuse [32]. The swelling capacity of the gel, therefore, plays an important role in determining the release profile of the system. Owing to the fact that the extent of swelling is largely affected by the amount of fluids the gel can take up upon hydration, the *WAR* value is adopted in this study as an indicator of the swelling capacity. The *WAR* value of the complex gel is found to be positively related to the mass percentage of CMC-Na (Figure 5A). Apart from the swelling behavior, the release profile of a gel depends predominately on the process of erosion, which is associated with material degradation resulted from crosslink dissolution and bond cleavage [33,34,35]. Susceptibility to erosion is shown to increase with the mass percentage of CMC-Na in the complex gel (Figure 5B).

### 3.3. Transdermal Delivery Performance of Vesicle-Loaded Complex Gels

**Figure 6:**
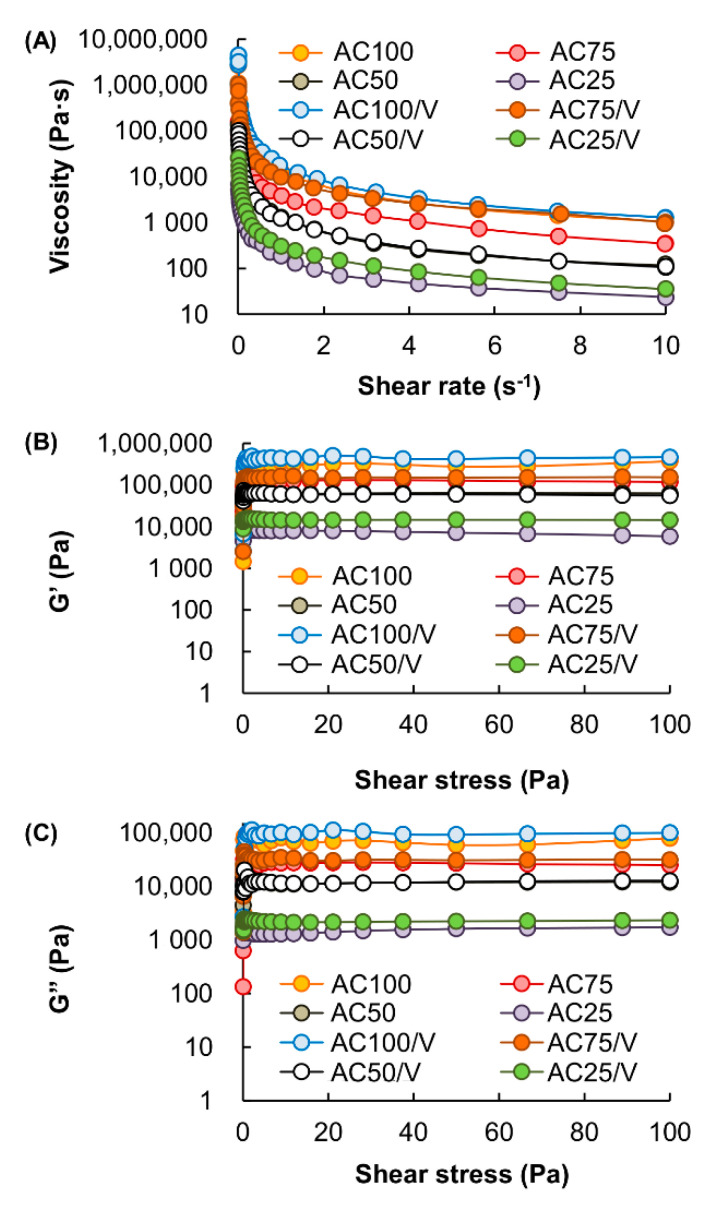
(**A**) Changes in the viscosity of different complex gels before and after being loaded with the vesicles. (**B**) Changes in the G′ values of different complex gels from 0 to 100 Pa. (**C**) Changes in the G″ values of different complex gels from 0 to 100 Pa.

To generate a vesicle-loaded complex gel, an aqueous solution containing both Na-Alg and CMC-Na is mixed with an aqueous solution of CaCl_2_ to initiate the gelation process. After the gel is lyophilized, a vesicle-containing solution is added to the lyophilized gel for vesicle loading. The apparent viscosity of all of the tested complex gels are higher at a low shear rate than at a high shear rate (Figure 6A). This is attributed to the pseudoplastic behavior of the gels. Such behavior remains upon the vesicle loading process. Changes in the G′ and G″ values under shear stress (Pa) at a constant frequency of 1 Hz are further examined by rheological analysis (Figure 6B,C). G′ values of all samples are higher than those of G″. This trend remains upon vesicle loading. This suggests that the tested complex gels display predominately the elastic character.

**Figure 7:**
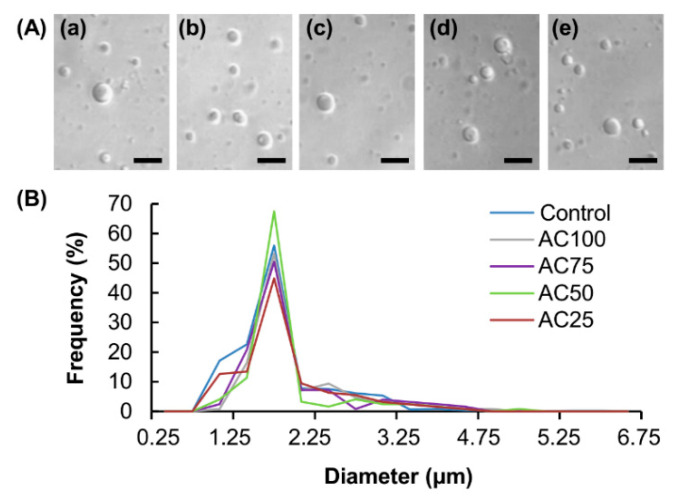
(**A**) Optical images of (**a**) freshly prepared blank vesicles and those released from (**b**) AC100, (**c**) AC75, (**d**) AC50 and (**e**) AC25. Scale bar = 10 μm. (**B**) The size distribution profiles of the blank vesicles upon being loaded into complex gels with different mass percentages of CMC-Na. Freshly prepared blank vesicles are adopted as the control.

**Figure 8:**
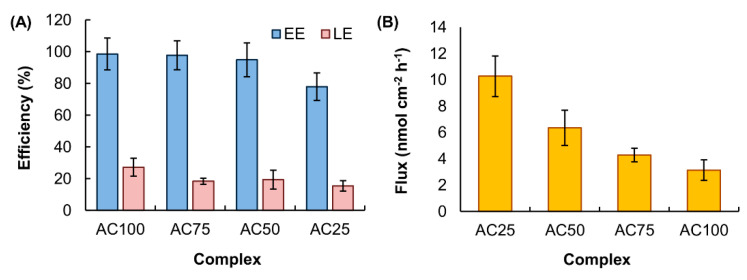
(**A**) The EE and LE of complex gels with different mass percentages of CMC-Na. Data are presented as the means ± SD of triplicate experiments. (**B**) The transdermal flux of THSG from different V-THSG-loaded complex gels. Data are presented as the means ± SD of triplicate experiments.

Compared with the size of freshly prepared blank vesicles, no significant change in the size of the vesicles is observed after being loaded into a complex gel (Figure 7). This suggests that the effect of the process of vesicle loading on the stability of the vesicles is negligible. There is no significant change in the EE and LE of the complex gels upon changes in the mass percentage of CMC-Na (Figure 8A); however, partly due to the fact that complex gels with higher mass percentages of CMC-Na show higher *WAR* values and erosion susceptibility, the release sustainability of the gels is expected to increase in the order: AC100 > AC75 > AC50 > AC25. The higher the release sustainability of the gel, the lower the transdermal flux of THSG from the gel per unit time. This explains the observation that when the mass percentage of CMC-Na in the complex gel increases, the transdermal flux of THSG from the V-THSG-loaded gel increases (Figure 8B).

## 4. Conclusions

Transdermal drug delivery is a non-invasive and user-friendly method for administration of therapeutic agents; however, partly due to the presence of the stratum corneum, which serves as a rate limiting barrier in transdermal permeation of most drugs [36], the practical use of transdermal drug delivery is impeded in the clinical context. From the data presented in this study, oleic acid-containing vesicles can effectively disrupt the integrity of the stratum corneum to enhance skin permeation of THSG, while showing good stability and negligible cytotoxicity. Upon the loading of the vesicles into a complex gel, which displays composition-dependent swelling and erosion behavior, a transdermal delivery system exhibiting tunable transdermal flux of THSG is obtained. It is hoped that with further optimization of the vesicle composition and with more detailed evaluation of the bioavailability of different drugs delivered by using the vesicles as carriers, a biocompatible, easy-to-make and tunable system can be made available for routine transdermal drug delivery in the future.
